# DGS1 improves rice disease resistance by elevating pathogen-associated molecular pattern-triggered immunity

**DOI:** 10.1007/s42994-024-00137-9

**Published:** 2024-02-06

**Authors:** Yu Wang, Chuan Zheng, You-liang Peng, Qian Chen

**Affiliations:** MOA Key Lab of Pest Monitoring and Green Management and Frontiers Science Center for Molecular Design Breeding, China Agricultural University, Beijing, 100193 China

**Keywords:** Rice blast, PTI, ERAD, E2–E3 pair, Ubiquitination

## Abstract

Rice yield and disease resistance are two crucial factors in determining the suitability of a gene for agricultural breeding. *Decreased grain size1* (*DGS1*), encoding an RING-type E3 ligase, has been found to have a positive effect on rice yield by regulating rice grain number and 1000-grain weight. However, the role of DGS1 in rice blast resistance is still unknown. In this study, we report that DGS1 enhances disease resistance by improving PTI responses, including stronger ROS burst and MAPK activation, and also increased expression of defense-related genes. Furthermore, DGS1 works in conjunction with ubiquitin conjugating enzyme OsUBC45 as an E2–E3 pair to facilitate the ubiquitin-dependent degradation of OsGSK3 and OsPIP2;1, thereby influencing rice yield and immunity, respectively. Therefore, the DGS1-OsUBC45 module has the potential in facilitating rice agricultural breeding.

**Supplementary Information:**

The online version contains supplementary material available at 10.1007/s42994-024-00137-9.

Dear Editor,

To improve global food security, enhancing rice yield is considered one of the most effective strategies. Rice yield is determined by several key components, including panicle number, grain number per panicle, and 1000-grain weight (Xing and Zhang 2010). Recently, a gene named *decreased grain size1* (*DGS1*), also known as *Thermo-tolerance 3.1* (*TT3.1*) and *Small Grain and Dwarf 1* (*SGD1*) in rice, was shown to have a positive effect on the regulation of rice grain number and 1000-grain weight, but not panicle number (Zhu et al. 2021; Zhang et al. 2022; Li et al. 2023). The *dgs1* mutants exhibited shorter panicles with fewer grain numbers per panicle and smaller grains, resulting in a more than 50% yield loss in rice, whereas *DGS1* overexpression lines showed longer grain length (Li et al. 2023). Notably, the role of *DGS1* on grain size regulation is conserved among Poaceae species, from maize, wheat, to millet (Tang et al. 2023). DGS1 encodes a RING-type E3 ligase containing a seven transmembrane Fragile-X-F domain and a C3HC4 type RING domain (Zhang et al. 2022; Tang et al. 2023). Researchers from two independent groups have shown that DGS1 cooperates with OsUBC45, an ERAD-related ubiquitin conjugating enzyme that is orthologous to AtUBC32 in *Arabidopsis*, to form an E2-E3 pair to control grain yield though regulating the accumulation of brassinosteroid (BR)-insensitive 1 (BRI1), the receptor of the phytohormone BR (Cui et al. 2012; Chen et al. 2021; Li et al. 2023; Tang et al. 2023). Thus, the *DGS1-OsUBC45-BRI* module plays an important role in improving grain yield.

In addition to grain yield, disease resistance is a crucial factor in determining the suitability of a gene for agricultural breeding. Our recent research has demonstrated that OsUBC45 enhances both rice yield and disease resistance by facilitating the 26S proteasome-dependent degradation of glycogen synthase kinase 3 (GSK3)/SHAGGY-like kinase 3 (OsGSK3) and aquaporin OsPIP2;1, which are negative regulators of grain size and pathogen-associated molecular pattern-triggered immunity (PTI), respectively (Gao et al. 2019; Wang et al. 2023). Similarly, SGD1 in millet has been shown to increase grain yield and resistance to blast disease. However, the mechanism underlying disease resistance remains unclear, although it is known to regulate yield through BR signaling (Tang et al. 2023). Furthermore, whether DGS1 in rice plays a role in blast resistance is also unknown. In this study, we report that the high yield player DGS1 also contributes to rice blast resistance. It works in conjunction with OsUBC45 to facilitate the ubiquitin-dependent degradation of OsGSK3 and OsPIP2;1, thereby influencing both rice yield and immunity. Collectively, the *DGS1*-*OsUBC45* gene module holds great potential as a target for rice breeding programs.

Firstly, to determine whether *DGS1* regulates rice disease resistance, similar to its collaborator OsUBC45, we compared the resistance of WT, two *dgs1* edited mutants and two overexpression lines to *M. oryzae* infection. In the wounding inoculation, *dgs1* formed significantly enlarged lesions and increased fungal biomass compared to WT, whereas the *DGS1* overexpression lines displayed smaller lesions and reduced fungal biomass (Fig. 1A, B and Supplemental Fig. 1). These findings indicate that DGS1 enhances resistance to rice blast. Additionally, since OsUBC45 enhances disease resistance by elevating PTI, we proceeded to perform several assays to determine whether DGS1 is involved in rice immunity through the PTI pathway. As depicted in Fig. 1C and D, the *DGS1* overexpression lines displayed stronger mitogen-activated protein kinase (MAPK) activation and a greater burst of reactive oxygen species (ROS) compared to WT plants, when subjected to chitin treatment. Furthermore, expression of defense-related genes *PR5-1* and *PR10* was upregulated in the overexpression lines compared to WT plants (Fig. 1E). Thus, overexpression of DGS1 in rice enhances disease resistance by improving PTI responses, in addition to positively impacting rice yield.

**Fig. 1:**
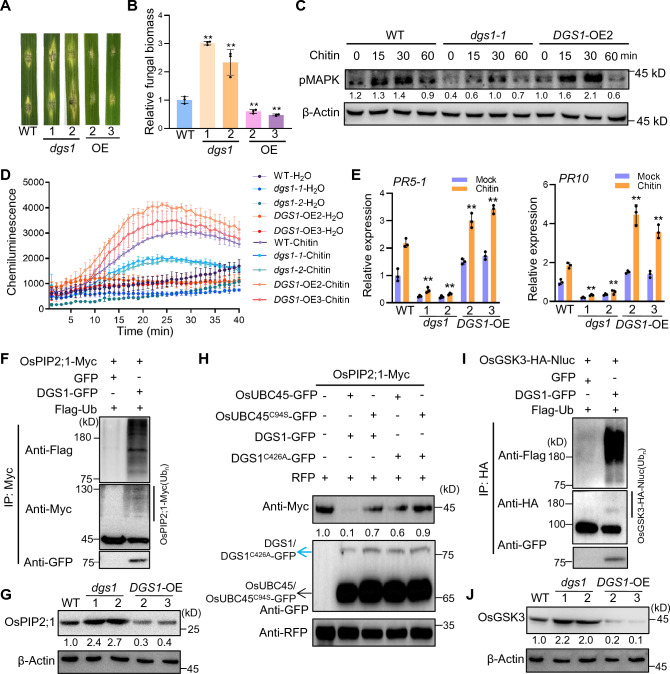
*DGS1* enhanced resistance to rice blast by positively regulating PTI. **A** *DGS1* positively regulated rice blast resistance. WT (ZH11), *dgs1* mutants and *DGS1* transgenic plants were inoculated with the *M. oryzae* virulent isolate. Leaves were photographed at 5 days-post-infection (dpi). **B** The relative fungal biomass (*n* = 3) was measured at 5 dpi. Error bars represent SEM; significant differences were evaluated by two-tailed Student’s *t* test analysis (**P < 0.01). **C** Chitin-induced MAPK activation in WT, *dgs1* mutants and *DGS1* transgenic plants. 10-day-old seedlings were treated with 10 μg/mL chitin and collected at 0, 15, 30, and 60 min. MAPK activation was detected by western blotting with an anti-phospho-p44/42 MAPK antibody. Actin was used as the internal control. **D** Chitin-induced ROS accumulation in WT, *dgs1* mutants and *DGS1* transgenic plants. ROS were determined using the luminol-based chemiluminescence assay. H_2_O treatment was used as the negative control. Error bars represent SEM, *n* = 3. **E** *DGS1* positively regulated the induction of the defense genes *PR5-1* and *PR10.* WT, *dgs1* mutants and *DGS1* transgenic seedlings were treated with 10 μg/mL chitin for 6 h. The expression of *PR5-1* and *PR10* was determined by qPCR. Error bars represent SEM, *n* = 3; significant differences were evaluated by two-tailed Student’s *t* test analysis (**P < 0.01). **F** DGS1 mediated ubiquitination of OsPIP2;1 in vivo. OsPIP2;1-Myc and Flag-ub were coexpressed with GFP or DGS1-GFP in rice protoplasts. The protoplasts were treated with 50 μM MG132 for 4 h before protein extraction. After being purified using anti-Myc magnetic beads, samples were detected using anti-Flag, anti-Myc and anti-GFP antibodies. **G** OsPIP2;1 was accumulated in the *dgs1* mutants and decreased in *DGS1*-OE plants. Protein levels of OsPIP2;1 in the WT, two *dgs1* gene edited lines and two *DGS1*-OE transgenic lines were measured using anti-OsPIP2;1 antibody. **H** The E3 ligase activity of DGS1 and E2 activity of OsUBC45 was important for the degradation of OsPIP2;1. Single amino acid mutations DGS1-C426A and OsUBC45-C94S had loss of E3 ligase and E2 activities, respectively. *Agrobacterium* with OsPIP2;1-Myc plasmid was co-injected into *N. benthamiana* with *Agrobacterium* containing the corresponding plasmids. The total protein of *N. benthamiana* leaves was extracted 2 days after infiltration and analyzed by western blot. RFP was used as the co-expression control. **I** DGS1 mediated ubiquitination of OsGSK3 in vivo*.* OsGSK3-HA-Nluc and Flag-ub were coexpressed with GFP or DGS1-GFP in rice protoplasts. The protoplasts were treated with 50 μM MG132 for 4 h before protein extraction. After being purified, using anti-HA agarose, samples were detected using anti-Flag, anti-HA and anti-GFP antibodies. **J** OsGSK3 was accumulated in the *dgs1* mutants and decreased in *DGS1*-OE plants. Protein levels of OsGSK3 in the WT, two *dgs1* gene edited lines and two *DGS1*-OE transgenic lines were measured with anti-OsGSK3 antibody

The study conducted by Wang et al. (2023) revealed that OsUBC45 enhanced rice yield and broad-spectrum disease resistance by targeting OsGSK3 and OsPIP2;1 for degradation. This finding prompted us to investigate whether OsPIP2;1 and OsGSK3 are also substrates of the E3 ligase DGS1. To address this question, we co-expressed OsPIP2;1-Myc with DGS1-GFP and Flag-Ub in rice protoplasts. Subsequently, we purified OsPIP2;1 using anti-Myc magnetic beads and assessed its ubiquitination level using an anti-Flag antibody. The results clearly demonstrated that DGS1 enhanced the ubiquitination of OsPIP2;1 (Fig. 1F). Additionally, we examined the impact of DGS1 on the stability of OsPIP2;1 using an anti-OsPIP2;1 antibody. Consistently, we observed higher levels of OsPIP2;1 in seedlings of *dgs1* mutants and significantly reduced levels in seedlings of the DGS1 overexpressing line, as compared to the WT (Fig. 1G). According to the cell-free or co-expression degradation assays *N. benthamiana*, DGS1-mediated degradation of OsPIP2;1 is time- and dose-dependent (Supplemental Fig. 2A and 2B). Furthermore, the proteasome inhibitor MG132 suppressed the DGS1-mediated degradation of OsPIP2;1 (Supplemental Fig. 2C). To determine the specificity of DGS1-OsUBC45 in the degradation of OsPIP2;1, we generated mutation constructs DGS1-C426A and OsUBC45-C94S, which abolish the E3 and E2 activities of DGS1/OsUBC45, respectively (Cui et al. 2012; Li et al. 2023). In the combined assay, coexpression of wild type DGS1/OsUBC45 led to a substantial reduction in the protein level of OsPIP2;1 (Fig. 1H). However, when using the active site mutated DGS1 or OsUBC45, or both, there was no significant change in the protein level of OsPIP2;1. These findings provide compelling evidence that OsPIP2;1 is indeed a substrate of the DGS1-OsUBC45 complex.

We also observed the degradation effect of DGS1 on OsGSK3. As depicted in Fig. 1I, after purification with anti-HA agarose beads, DGS1 significantly increased the ubiquitination level of OsGSK3. Additionally, we assessed the protein levels of OsGSK3 in seedlings of *DGS1* overexpression and edited lines. The protein levels of OsGSK3 were notably reduced in *DGS1* overexpression lines but accumulated more in the *dgs1* mutants compared to WT (Fig. 1J). This result indicated that DGS1 negatively regulates the stability of OsGSK3 through enhancing its ubiquitination. Similar to OsPIP2;1, the degradation of OsGSK3 is enhanced with higher doses and longer exposure to DGS1 (Supplemental Fig. 2D and 2E). At the same time, the degradation of OsGSK3, mediated by DGS1, can be prevented by MG132 (Supplemental Fig. 2F). Consistently, the combined assay with OsGSK3 showed that only the wild type DGS1-OsUBC45 could promote its degradation, whereas DGS1C426A-OsUBC45C94S could not (Supplemental Fig. 3). Consequently, DGS1 and OsUBC45 function as an E2–E3 pair to mediate the ubiquitination and degradation of both OsPIP2;1 and OsGSK3.

Taken together, our data establish that the collaboration between DGS1 and OsUBC45, as an E2–E3 pair, plays a crucial role in the ubiquitination process of OsGSK3 and OsPIP2;1, which may thereby regulate rice yield and immunity, respectively. Previous studies have demonstrated that DGS1, in conjunction with OsUBC45, can regulate grain size by enhancing the accumulation of BRI1, a positive regulator of BR signaling. However, our study suggests that DGS1 may also regulate grain size by promoting the degradation of OsGSK3, a negative regulator in BR signaling. Therefore, we propose that the DGS1-OsUBC45 module improves rice yield by regulating various components in the BR signaling pathway. Additionally, our findings indicate that DGS1 enhances rice blast resistance by positively regulating PTI responses, including increased expression of PR genes, stronger ROS burst, and MAPK activation (Supplemental Fig. 4). Overall, the *DGS1*-*OsUBC45* module holds great potential for enhancing rice agricultural breeding programs.

## Materials and methods

### Pathogen inoculation

To inoculate *M. oryzae*, the second newly expanded leaves of rice plants at the four- to eight-leaf stage were wounded and exposed to *M. oryzae* spores at a concentration of 1.5 × 10^5^ spores/mL. The inoculated plants were then transferred to darkness at 28 °C for 24 h, followed by a growth period of 14 h of light and 10 h of darkness for disease symptom development. Disease symptoms were recorded at 5 days after inoculation (dpi). The size of the disease lesions was measured using Image J software. To determine the relative *M. oryzae* biomass, DNA-based qPCR was used to calculate the ratio of *M. oryzae MoPot2* DNA to rice *OsUBQ10* DNA.

### RT-qPCR

Total RNA was isolated by KK Fast Plant Total RNA Kit (Beijing Zoman Biotechnology) according to the manufacturer’s protocol and was reverse-transcribed by HiScript III 1st Strand cDNA Synthesis Kit (Vazyme Biotech Co., Ltd., Nanjing, China) with gDNA. *OsUBQ10* was used as internal control for qPCR. Gene-specific primers are presented in Supplementary Table 1.

### Rice protoplast preparation and transfection

2-week-old etiolated seedlings grown on 1/2 MS medium in the dark at 23 °C in an incubator were used for this experiment. The seedlings were cut into 0.5 mm strips and incubated in a solution containing 1.5% Cellulase R-10, 0.75% Macerozyme, 0.6 M D-mannitol, 10 mM MES, 1 mM CaCl_2_, and 50 mM β-mercaptoethanol (pH 5.7). This incubation step lasted for 4–6 h in the dark at room temperature with gentle shaking. After incubation, the strips were washed twice with W5 solution [154 mM NaCl, 125 mM CaCl_2_, 5 mM KCl, 2 mM MES (pH 5.7)] at room temperature and filtered through a Miracloth layer. The resulting protoplasts were collected by centrifugation at 300 g for 5 min, washed once with W5 solution, and suspended at a concentration of 2.0 × 10^6^ cells/mL.

For each transfection reaction, 5 μg plasmid was added to 300 μL protoplasts and incubating at room temperature for 15 min. The transfected protoplasts were washed once with W5 solution and kept in the dark at room temperature for 18 h before analysis. Total protein was extracted using a buffer containing 50 mM Tris–HCl (pH 8.0), 0.5 M sucrose, 1 mM MgCl_2_, 10 mM EDTA, 5 mM DTT, and protease inhibitor cocktail Complete Mini tablets (Roche).

### In vivo ubiquitination assay

For the in vivo ubiquitination assay, OsGSK3-HA-Nluc or OsPIP2;1-Myc was co-expressed with DGS1-GFP and Flag-ubiquitin in rice protoplast cells. OsGSK3-HA-Nluc or OsPIP2;1-Myc proteins were extracted after being treated with 50 μM MG132 (133407-82-6, MCE) for 4 h. Samples were purified using anti-HA nanoab-agarose (HNA-25-500, LABLEAD) or Myc-tagged mAb-magnetic agarose (M047-10-400UL, MBL) respectively. The protein ubiquitination level of OsGSK3-HA-Nluc or OsPIP2;1-Myc were analyzed using anti-Flag antibody.

### Protein degradation assays in *Nicotiana benthamiana* and rice protoplasts

For the combined degradation assays of OsUBC45/DGS1, different *Agrobacterium tumefaciens* cultures harboring OsPIP2;1-Myc (or OsGSK3-HA-Nluc) was mixed with equal volumes of *A. tumefaciens* harboring OsUBC45/DGS1, and co-expressed in *N. benthamiana*. For the gradient degradation assays, OsPIP2;1-Myc (or OsGSK3-HA-Nluc) was coexpressed with different dose of DGS1-GFP in *N. benthamiana*. Two days later, total proteins were extracted and then detected using the corresponding antibodies.

For the time course degradation assay, DGS1-GFP, GFP, OsPIP2;1-Myc and OsGSK3-HA-Nluc were expressed, respectively in *N. benthamiana*. The proteins were extracted and mixed according to the need. The mixtures were subjected to immunoblotting with an anti-Myc or anti-HA antibody after being incubated at 25 °C for different time.

For the degradation performed in rice protoplasts, GFP or DGS1-GFP plasmids were coexpressed with OsPIP2;1-Myc plasmid (or OsGSK3-HA-Nluc plasmid) in rice protoplasts. The protoplasts were treated with or without 50 μM MG132 for 4 h before being collected. The proteins were extracted and then detected using corresponding antibodies.

### MAPK assay

For MAPK assays, sterile rice seedlings were treated with 10 μg/mL chitin (C9752-5G, Sigma) and collected at different time points. The total proteins were extracted using an extraction buffer that included a phosphatase inhibitor. Subsequently, immunoblotting analysis was performed to detect phosphorylated MAPK proteins using an anti phospho-p44/42 antibody (9101S-200UL, Cell Signaling).

### ROS detection

For ROS burst, the rice leaves were cut into a diameter of 4 mm disks. Each sample were floated on ddH_2_O for overnight recovery. Before detection, the water was replaced with solution containing 0.02 mM luminol and 20 μg/mL horseradish peroxidase with or without 10 μg/mL chitin. Luminescence was measured continuously by TECAN Infinite^®^ F200 microplate reader (TECAN, Switzerland).

### Accession numbers

Sequence data from this article can be found in the rice genome annotation project database under the following accession numbers: *OsUBC45*, LOC_Os03g19500; *DGS1*, LOC_Os03g49900; *OsPIP2;1*, LOC_Os07g26690 and *OsGSK3*, LOC_Os02g14130.

### Antibodies

Anti-GFP (BE2001-100UL, EASYBIO), anti-Myc (BE2010-100UL, EASYBIO), anti-HA (BE2071-100UL, EASYBIO), anti-Actin (BE0027-100UL, EASYBIO), anti-PIP2;1 (AS194328-100UL, Agrisera), anti-Flag (BE2004-100, EASYBIO) and anti-RFP (BE2023-100UL, EASYBIO) antibodies were used to bind the protein with the indicated tag. Secondary peroxidase-conjugated anti-mouse (BE0120-100UL, EASYBIO) or anti-rabbit (SA00001-2-100UL, Proteintech) antibody was used at a 1:5000 dilution.

### Statistical analysis

Statistical analyses were performed using GraphPad Prism 9. Image analyses were generated from ImageJ. qPCR, fungal biomass, and ROS assays were usually calculated from three replicates, and data are presented as means ± SEM. Statistically significant differences were determined by two-tailed Student’s *t* test: *P < 0.05 and **P < 0.01.

### Supplementary Information

Below is the link to the electronic supplementary material.

Supplementary file1 (PDF 911 kb)

## Data Availability

The data that support the findings of this study are available from the corresponding author upon reasonable request.
