# Supplementary material for: DGS1 improves rice disease resistance by elevating pathogen-associated molecular pattern-triggered immunity
Source: aBIOTECH. 2024 Feb 6;5(1):46–51. doi: 10.1007/s42994-024-00137-9 (PMC10987426; doi:10.1007/s42994-024-00137-9)
Supplement: Supplementary file 1 — Supplementary file1 (PDF 911 kb) [file 42994_2024_137_MOESM1_ESM.pdf]

Wang et al., Figure 1

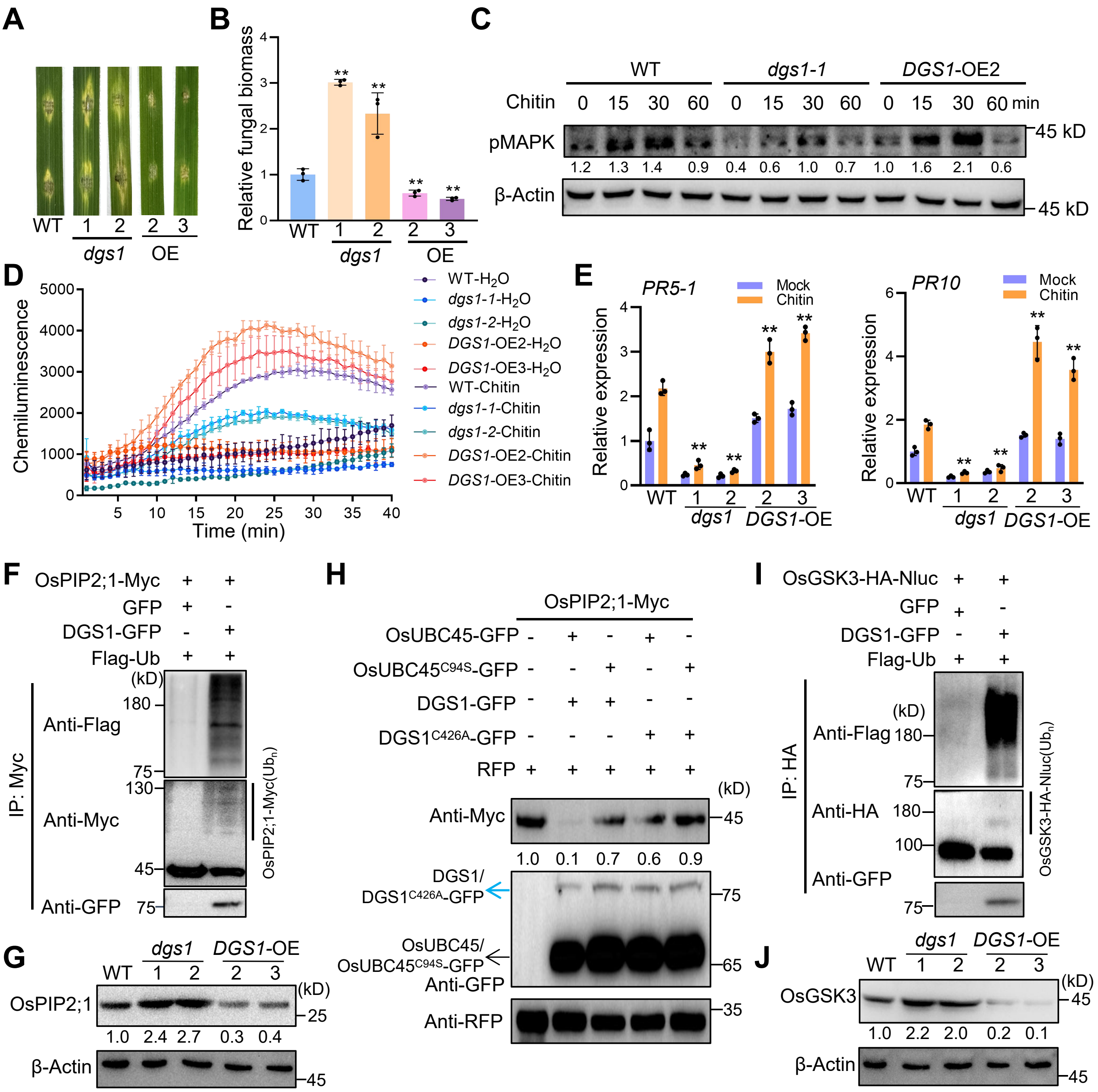

Fig. 1 *DGS1* enhanced resistance to rice blast by positively regulating PTI. **A** *DGS1* positively regulated rice blast resistance. WT (ZH11), *dgs1* mutants and *DGS1* transgenic plants were inoculated with the *M. oryzae* virulent isolate. Leaves were photographed at 5 dpi. **B** The relative fungal biomass (n = 3) was measured at 5 dpi. Error bars represent SEM. The significant differences were evaluated by two-tailed Student's t-test analysis (**$p < 0.01$). **C** Chitin-induced MAPK activation in WT, *dgs1* mutants and *DGS1* transgenic plants. Ten-day-old seedlings were treated with 10 µg/mL chitin and collected at 0, 15, 30, and 60 mins. MAPK activation was detected by western blotting with an anti-phospho-p44/42 MAPK antibody. Actin was used as the internal control. **D** Chitin-induced ROS accumulation in WT, *dgs1* mutants and *DGS1* transgenic plants. ROS were determined using the luminol-based chemiluminescence assay. H_2O treatment was used as the negative control. Error bars represent SEM, n=3. **E** *DGS1* positively regulated the induction of the defense genes *PR5-1* and *PR10.* WT, *dgs1* mutants and *DGS1* transgenic seedlings were treated with 10 µg/mL chitin for 6 h. The expression of *PR5-1* and *PR10* was determined by qPCR. Error bars represent SEM, n = 3. The significant differences were evaluated by two-tailed Student's t-test analysis (**$p < 0.01$). **F** DGS1 mediated ubiquitylation of OsPIP2;1 *in vivo*. OsPIP2;1-Myc and Flag-ub were coexpressed with GFP or DGS1-GFP in rice protoplasts. The protoplasts were treated with 50 µM MG132 for 4 h before protein extraction. After being purified using anti-Myc magnetic beads, samples were detected using anti-Flag, anti-Myc and anti-GFP antibodies. **G** OsPIP2;1 was accumulated in the *dgs1* mutants and decreased in *DGS1*-OE plants. Protein levels of OsPIP2;1 in the WT, two *dgs1* gene edited lines and two *DGS1*-OE transgenic lines were measured using anti-OsPIP2;1 antibody. **H** The E3 ligase activity of DGS1 and E2 activity of OsUBC45 were important for the degradation of OsPIP2;1. Single amino acid mutations DGS1-C426A and OsUBC45-C94S lost E3 ligase activity and E2 activity, respectively. *Agrobacterium* with OsPIP2;1-Myc plasmid was co-injected into *N. benthamiana* with *Agrobacterium* containing the corresponding plasmids. The total protein of *N. benthamiana* leaves was extracted 2 days after infiltration and analyzed by western blot. RFP was used as the co-expression control. **I** DGS1 mediated ubiquitylation of OsGSK3 *in vivo.* OsGSK3-HA-Nluc and Flag-ub were coexpressed with GFP or DGS1-GFP in rice protoplasts. The protoplasts were treated with 50 µM MG132 for 4 h before protein extraction. After being purified using anti-HA agrose, samples were detected using anti-Flag, anti-HA and anti-GFP antibodies. **J** OsGSK3 was accumulated in the *dgs1* mutants and decreased in *DGS1*-OE plants. Protein levels of OsGSK3 in the WT, two *dgs1* gene edited lines and two *DGS1*-OE transgenic lines were measured with anti-OsGSK3 antibody.

Supplemental Figure 1

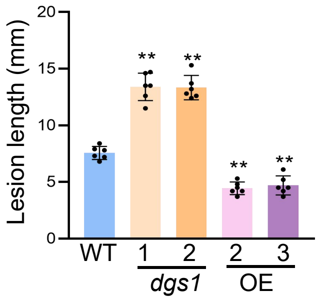

Supplemental Fig. 1 Lesion length of the drop inoculation in Fig. 1 (A) (n = 6). Error bars represent SEM. The significant differences were evaluated by two-tailed Student's t-test analysis (**$p < 0.01$).

Supplemental Figure 2

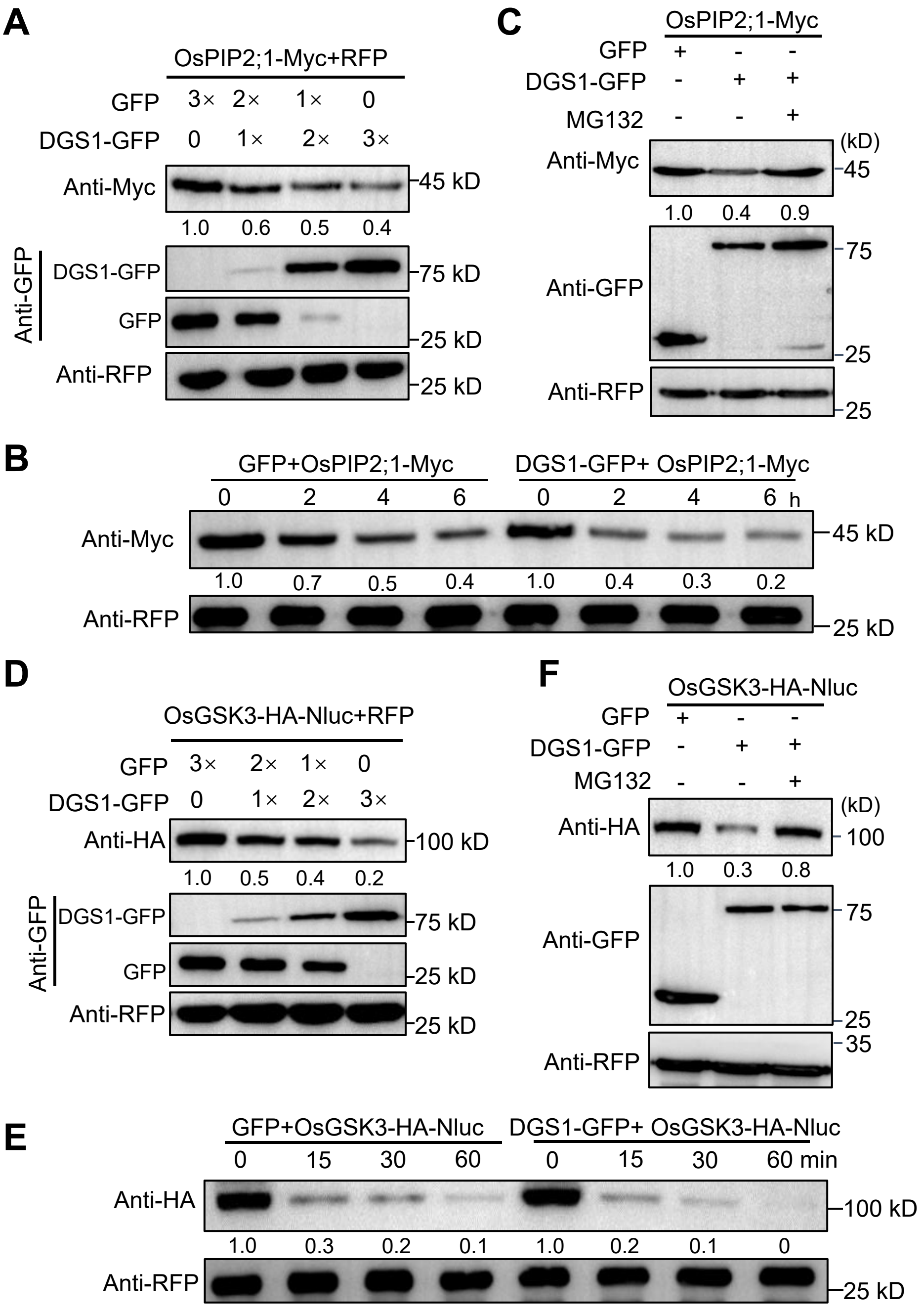

Supplemental Fig. 2 DGS1 promoted the degradation of OsPIP2;1 and OsGSK3. **A** and **D** OsPIP2;1-Myc or OsGSK3-HA-Nluc and DGS1-GFP were co-expressed in *N. benthamiana*, respectively. After two days, total proteins were extracted and detected using the appropriate antibodies. **B** and **E** DGS1-mediated the degradation of OsPIP2;1 and OsGSK3 is time-dependent. The proteins were expressed separately in *N. benthamiana* and the extracted proteins were mixed and incubated at 25°C for varying durations. The mixtures were then detected using anti-Myc or anti-HA antibody. **C** and **F** GFP or DGS1-GFP plasmids were coexpressed with OsPIP2;1-Myc plasmid (or OsGSK3-HA-Nluc plasmid) in rice protoplasts. The protoplasts were treated with DMSO or 50 μM MG132 for 4 h before protein extraction. The proteins were detected using corresponding antibodies.

Supplemental Figure 3

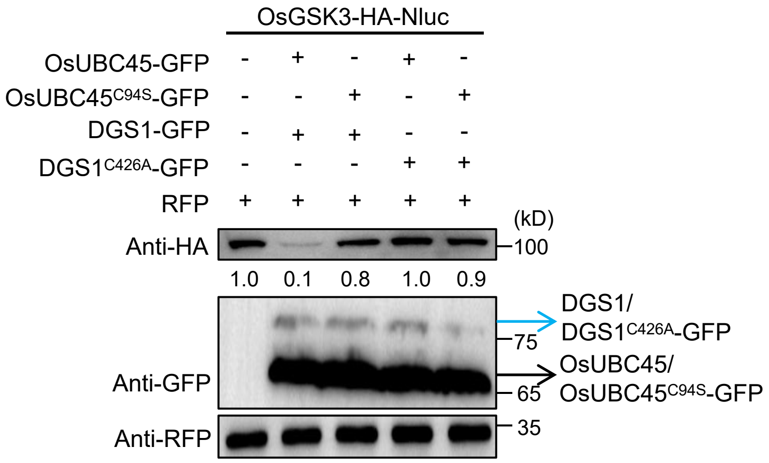

Supplemental Fig. 3 The E3 ligase activity of DGS1 and E2 activity of OsUBC45 were important for the degradation of OsGSK3. Single amino acid mutations DGS1-C426A and OsUBC45-C94S lost the E3 ligase activity and E2 activity, respectively. *Agrobacterium* with OsGSK3-HA-Nluc plasmid was co-injected into *N. benthamiana* with *Agrobacterium* containing the corresponding plasmid. The total protein of *N. benthamiana* leaves was extracted 2 days after infiltration and analyzed by western blot. RFP was used as the co-expression control.

Supplemental Figure 4

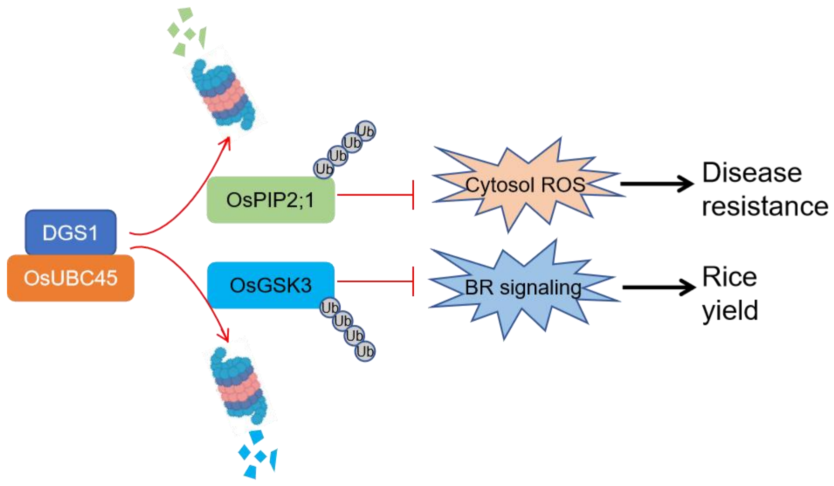

Supplemental Fig. 4 A proposed working model for OsUBC45-DGS1 pair in the plant response to regulate rice blast resistance and yield.

Supplemental Table 1

Primers for qPCR	
qPR5-1-F	CGGCAGCCAGGACTTCTA
qPR5-1-R	GCAGAAGACGACTTGGTAG
qPR10-F	GTCCGGGCACCATCTACACC
qPR10-R	CAAGCTTCGTCTCCGTCGAGT
qMoPOT-F	ACGACCCGTCTTTACTTATTTGG
*qMoPO*T-R	AAGTAGCGTTGGTTTTGTTGGAT
qgUBQ-F	TTCTGGTCCTTCCACTTTCAG
qgUBQ-R	ACGATTGATTTAACCAGTCCATGA
qOsUBQ10-F	TGGTCAGTAATCAGCCAGTTTGG
qOsUBQ10-R	GCACCACAAATACTTGACGAACAG
Primers for vector construction	
OsUBC45-GFP-F	CATGGTACC ATGGAGGCCACGGCGAAGTA
OsUBC45-GFP-R	TCCTCTAGA AAACTTGCCCTCAATGTAACC
OsUBC45-C94S-F	GAGATTCAAAAGAAGATTAGTTTGAGCATATCCA
OsUBC45-C94S-R	TGGATATGCTCAAACTAATCTTCTTTTGAATCTC
DGS1-GFP-F	GGACGAGCTCGGTACC ATGCAGCGGCGGCGGGCG
DGS1-GFP-R	GTGTCGACTCTAGA AACATCATATACGGGCATGCG
DGS1-C426A-F	AGGTTCTTGCTAGGATTTGC
DGS1-C426A-R	GCAAATCCTAGCAAGAACCT
OsPIP2;1-Myc-F	TCTGAAGAGGACTTGAATTCGGTACCC ATGGGGAAGGACGAGGTGATG
OsPIP2;1-Myc-R	ACGCGTCCTAGGCTACGTAGGATCC TCACGCGTTGCTCCTGAAGG
OsGSK3-HA-Nluc-F	ACGGGGGACGAGCTCGGTACC ATGGCCACGCTGCCGGGC
OsGSK3-HA-Nluc-R	AACATCGTATGGGTAGTCGAC CCGAGCATGCTCTGGTATCAA
